# A Case Series of Ketoacidosis After Coronavirus Disease 2019 Vaccination in Patients With Type 1 Diabetes

**DOI:** 10.3389/fendo.2022.840580

**Published:** 2022-03-18

**Authors:** Fumiyoshi Yakou, Masuo Saburi, Ai Hirose, Hiroaki Akaoka, Yusuke Hirota, Takaaki Kobayashi, Naoko Awane, Nobuteru Asahi, Toshihiro Amagawa, Sachihiko Ozawa, Atsushi Ohno, Takaya Matsushita

**Affiliations:** ^1^ Department of Diabetology, Endocrinology and Metabolism, Tokyo Medical University Hachioji Medical Center, Hachioji, Japan; ^2^ Minamino Tou Clinic, Hachioji, Japan

**Keywords:** coronavirus disease 2019 vaccination, ketoacidosis, type 1 diabetes, sodium-glucose cotransporter 2 inhibitor, clinical diabetes, latent autoimmune diabetes in adults

## Abstract

**Introduction:**

We report a case series of severe ketoacidosis after COVID-19 vaccination in a type 1 diabetes patients treated with insulin and an SGLT-2 inhibitor.

**Case Report:**

We present two cases of type 1 diabetes mellitus. One patient was treated with insulin therapy and an SGLT-2 inhibitor, and the other patient was treated with insulin therapy alone. Both patients became ill after coronavirus disease-2019 vaccination, making it difficult to continue their diet or insulin injections. On admission, they developed severe diabetic ketoacidosis. This is the first report of ketoacidosis after coronavirus disease-2019 vaccination.

**Conclusion:**

The vaccine should be carefully administered to type 1 diabetes patients receiving intensive insulin therapy and a sodium-glucose transporter due to the high risk ketoacidosis. It is important to instruct patients to drink sufficient fluids and to continue insulin injections when they become sick.

## Highlights

→In patients with type 1 diabetes, COVID-19 vaccination can trigger illness and may be a clinical challenge that requires prompt treatment.
→Prior to COVID-19 vaccination, patients with type 1 diabetes may need to be assured of what to do if they become sick.
→Please instruct the patient to drink sufficient fluids and continue insulin injections. This is posted as a message for patients on the ADA homepage (https://www.diabetes.org/coronavirus-covid-19/know-what-to-do).

## Introduction

COVID-19 infection can have serious consequences for people suffering from diabetes, obesity, malnutrition, Cushing’s syndrome (CS), and adrenal insufficiency (1). The coronavirus disease 2019 (COVID-19) vaccine is expected to reduce the incidence and risk of severe disease among patients with diabetes and elderly individuals, who are particularly predisposed to critical illness from COVID-19 infection (2).

On the other hand, the COVID-19 vaccine is reportedly associated with nausea and vomiting as adverse effects (3, 4). Loss of appetite and prolonged general malaise are likely to cause insulin injection failure. The interruption of treatment with insulin injections has reportedly induced diabetic ketoacidosis in type 1 diabetes mellitus patients. In addition, treatment with sodium-glucose transporter 2 (SGLT-2) inhibitors may increase the risk of developing diabetic ketoacidosis in patients with type 1 diabetes (5).

We encountered two cases of severe ketoacidosis associated with COVID-19 vaccination in type 1 diabetes patients. We present those cases because there have been no prior reports on the occurrence of this complication.

## Case Report

### First Case

A 71-year-old woman was diagnosed with diabetes mellitus at 56 years of age. She had a history of hyperthyroidism due to Basedow disease when she was 52 years of age. After three years of anti-thyroid therapy, her hyperthyroidism went into remission. She received oral drugs after being diagnosed with diabetes mellitus at 56 years of age.

Seven months later, blood examination revealed an elevated level of anti-glutamic acid decarboxylase antibody (869 U/ml). She then received insulin injection therapy for type 1 diabetes mellitus. Her glycated hemoglobin (HbA1c) level was approximately 8%, and she had no history of ketosis or ketoacidosis.

She was treated with insulin glulisine (13 units/day, pre-breakfast 4 units, pre-lunch 4 units, pre-dinner 5 units), insulin degludec (pre-dinner 3 units), and a SGLT-2 inhibitor, ipragliflozin (25 mg/day). Her fasting blood glucose levels in the morning before COVID-19 vaccination ranged from 93 to 169 mg/dl. Her HbA1c levels at approximately three months and one month before admission were 8.3% and 8.1%, respectively. She received the Pfizer-BioNTech COVID-19 (BNT162b2) vaccine (Pfizer, Inc; Philadelphia, Pennsylvania) three days prior to admission. Immediately after COVID-19 vaccination, she developed nausea and fatigue, and her water and dietary intake decreased. From the next day, the patient showed impaired consciousness, which made it impossible for her to inject insulin or take SGLT-2 inhibitor. Her symptoms worsened, and she was taken to the emergency room of Tokyo Medical University Hachioji Medical Center, Hachioji, Tokyo, Japan.

On arrival, she had tachycardia and tachypnea, and her Glasgow Coma Scale was 12 (E3V4M5). A laboratory evaluation showed severe acidosis, ketonuria, ketonemia (**Table 1**). Chest X-ray, electrocardiography, abdominal computed tomography, and urinary sediments showed no abnormalities. Thus, an infectious disease was unlikely. The patient was diagnosed with ketoacidosis. Immediately after admission, continuous intravenous insulin, Ringer’s solution, and glucose infusion were initiated in the intensive care unit.

**Table 1:** The patient’s physical examinations and laboratory data on admission.

Physical Examination					
BH 151.8 cm, BW 41.4 kg, BMI 18.0 kg/m^2^, RR 24 times/min, BP 118/76 mmHg, Glasgow Coma Scale E3V4M5					
Complete Blood Count		Urinalysis		Arterial Blood Gas Analysis	
White blood cell count	15300 /μL	Protein	2+	pH	7.049
Neutrophil	72.8%	Glucose	4+	pCO_2_	12.4 mmHg
Lymphocyte	15.7%	Ketone body	1+	pO_2_	128 mmHg
Red blood cell count	405×10^4^ /μL	Urinary osmotic pressure	360 mOsm/L	HCO_3_^-^	3.3 mmol/L
Hemoglobin	13.3 g/dL	C-peptide (urine)	<0.8 µg/day	Base excess	-26.9 mmol/L
Hematocrit	44%			Lactate	26 mg/dL
Platelet count	17.4×10^4^ /μL			Anion gap	28.9 mmol/L
**Blood chemistry**					
Aspartate aminotransferase	67 IU/L	Sodium	118 mEq/L	Glycoalbumin	27.7%
Alanine aminotransferase	34 IU/L	Potassium	6.7 mEq/L	C-peptide (serum)	<0.03 ng/mL
Lactate dehydrogenase	282 IU/L	Chlorine	79 mEq/L	anti-GAD antibody	>2000 U/mL
Total protein	6.6 g/dL	Calcium	8.4 mg/dL	Islet antigen 2 antibody	N.D
Albumin	4 g/dL	Phosphate	9 mg/dL	Anti-insulin autoantibody	N.D
Total bilirubin	0.6 mg/dL	Magnesium	2.4 mg/dL	Total ketone body	16427 μmol/L
Amylase	9696 U/L	Uric acid	11.7 mg/dL	Acetoacetate	3340 μmol/L
p-Amylase	304 U/L	Blood urea nitrogen	90.9 mg/dL	3-Hydroxybutyrate	13087 μmol/L
Lipase	13 U/L	Serum Creatinine	1.93 mg/dL	TSH	1.01 μU/mL
Trypsin	219 ng/mL	Fasting blood glucose	938 mg/dL	freeT3	1.94 pg/mL
Osmotic pressure	344 mOsm/L	HbA1c	8%	freeT4	1.34 ng/dL
**Past Disease History except diabetes**					
She was diagnosed with Basedow disease at the age of 52, but is now in remission. There was no history such as hypertension or cardiovascular disease.					

N.D, not detected.

**Figure 1:**
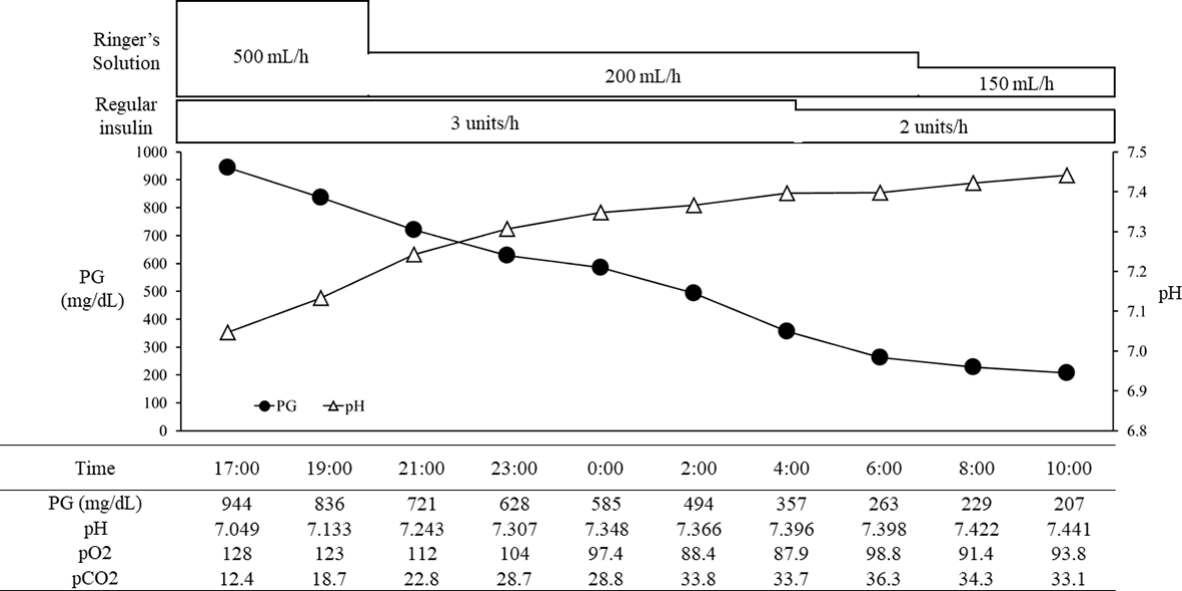
The course in day1-2 in a patient with ketoacidosis who was treated with insulin and SGLT2 inhibitor. PG, plasma glucose.

With the above combined treatment, her ketoacidosis gradually improved (**Figure 1**). On admission, the plasma glucose, HbA1c, and glycated albumin levels were 944 mg/dL, 8.0%, and 27.7%, respectively. She tested positive for anti-glutamic acid decarboxylase antibody, but was negative for insulinoma associated antigen-2 antibody and insulin autoantibody (**Table 1**). Serum C-peptide and urinary C-peptide were undetectable. After her ketoacidosis improved, intensive insulin therapy without SGLT-2 inhibitor was resumed. Serum C-peptide was also below the limit of detection in a glucagon load test (**Supplemental Figure 1**). When the patient was discharged, she was treated with insulin glulisine (17 units/day, pre-breakfast 7 units, pre-lunch 6 units, pre-dinner 4 units) and insulin degludec (pre-dinner 4 units). 

### Second Case

The patient was a 52-year-old woman who received insulin infusion therapy with multiple daily injections after being diagnosed with type 1 diabetes mellitus at 47 years of age. At the time of the diagnosis, she was admitted to another facility due to diabetic ketosis. She noticed thirst and >10 kg body weight loss in comparison to 6 months before her admission. A blood examination showed an elevated HbA1c level (15.5%), an elevated anti-glutamic acid decarboxylase antibody level (1230 U/ml), and an elevated islet antigen 2 antibody level (or*insulinoma-associated protein-2 antibody) (>41 U/mL). The urinary c-peptide level (45 µg/day) indicated that her insulin secretory capacity remained. She was diagnosed with latent autoimmune diabetes in adults and insulin treatment was continued.

She was treated by a family doctor with insulin aspart (25 units/day, pre-breakfast 11 units, pre-lunch 8 units, pre-dinner 6 units) and insulin degludec (before sleeping 12 units). However, the transition of her HbA1c level was poor (10-11%). Her fasting blood glucose levels in the morning before COVID-19 vaccination ranged from 106 to 262 mg/dl. She received her second vaccination the day before admission. For both vaccinations, she received the Pfizer-BioNTech COVID-19 (BNT162b2) vaccine (Pfizer, Inc; Philadelphia, Pennsylvania). She had a drinking habit and consumed approximately 20 g of alcohol on the night before vaccination.

Immediately after the second COVID-19 vaccination, she had symptoms of nausea, palpitation and respiratory distress. Her symptoms worsened, she could not inject insulin or eat from noon on that day. The following day she was taken to the emergency room of Tokyo Medical University Hachioji Medical Center, Hachioji, Tokyo, Japan.

**Figure 2:**
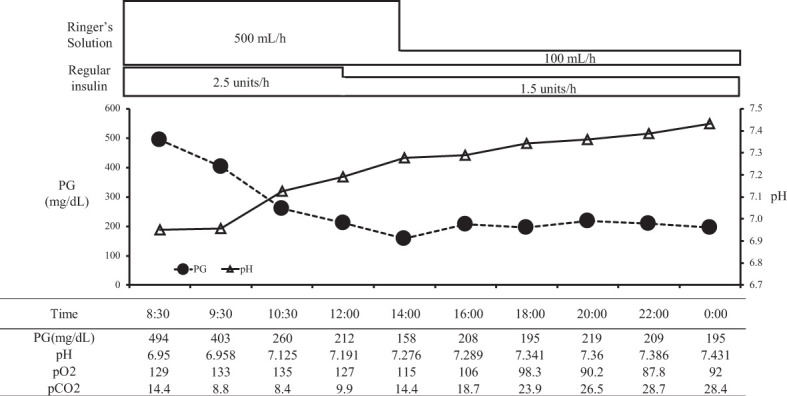
The course in day1-2 in a patient with ketoacidosis who was treated with insulin. PG, plasma glucose.

On arrival, she had tachycardia and tachypnea, but her consciousness was clear (E4V5M6). A laboratory evaluation showed severe acidosis, ketonuria, and ketonemia (**Table 2**). The patient was diagnosed with diabetic ketoacidosis. Immediately after admission, continuous intravenous insulin, Ringer's solution, and glucose infusion were initiated in the intensive care unit. With these combined treatments, her ketoacidosis gradually improved (**Figure 2**). On admission, her plasma glucose and HbA1c values were 494 mg/dL and 11.6%, respectively (**Table 2**). Serum C-peptide and the urinary C-peptide were slightly detected. Her thyroid hormone levels were normal. After her ketoacidosis improved, intensive insulin therapy was resumed.

**Table 2:** The patient's physical examinations and laboratory data on admission.

Physical examination					
BH 162 cm, BW 60.3 kg, BMI 23.0 kg/m^2^, RR 36 times/min, BP 168/82 mmHg, Glasgow Coma Scale E4V5M6					
Complete Blood Count		Urinalysis		Arterial Blood Gas Analysis	
White blood cell count	24600 /μL	Protein	1+	pH	6.958
Neutrophil	88.3%	Glucose	4+	pCO_2_	8.8 mHg
Lymphocyte	3.0%	Ketone body	4+	pO_2_	133 mmHg
Red blood cell count	599x10^4^ / μL	Urinary osmotic pressure	1.3 μg/day	HCO_3_^-^	1.9 mmol/L
Hemoglobin	13.9 g/dL			Base excess	-30.4 mmol/L
Hematocrit	45%			Lactate	16 mg/dL
Platelet count	45.8x10^4^ / μL			Anion gap	25.9 mmol/L
**Blood chemistry**					
Aspartate aminotransferase	15 IU/L	Sodium	129 mEq/L	Fasting blood glucose	494 mg/dL
Alanine aminotransferase	12 IU/L	Potassium	6.7 mEq/L	HbA1c	11.6%
Lactate dehydrogenase	361 IU/L	Chlorine	94 mEq/L	Glycoalburnin	N.D
Total protein	9.2 g/dL	Calcium	9.4 mg/dL	C-peptide (serum)	N.D
Albumin	4.6 g/dL	Phosphate	5.3 mg/dL	anti-GAD antibody	123 U/mL
Total bilirubin	0.3 rng/dL	Magnesium	N.D	Islet antigen 2 antibody	N.D
Amylase	112 U/L	Uric acid	N.D	Anti-insulin autoantibody	N.D
		Blood urea nitrogen	17.5 mg/dL	Total ketone body	13856 μmol/L
		Serum Creatinine	0.64 mg/dL	Acetoacetate	3275 μmol/L
				3-Hydroxybutyrate	10581 μmol/L
**Past Diseases History except diabetes**					
She has no history such as blood pressure or cardiovascular disease					

N.D, not detected.

When the patient was discharged, she was treated with insulin glulisine (27 units/day, pre-breakfast 11 units, pre-lunch 8 units, pre-dinner 8 units) and insulin degludec (before sleeping 13 units).

## Discussion

We reported two cases of ketoacidosis that occurred after COVID-19 vaccination in patients with latent autoimmune diabetes in adults. The first patient was diagnosed with diabetic ketoacidosis based on a blood glucose level of 944 mg/dL, arterial pH of 7.049, serum bicarbonate of 3.3 mEq/L, and urinal ketone body positivity on admission. Because the patient did not consume alcohol or excessive soft drinks after vaccination, it was considered unlikely that her ketoacidosis was caused by alcohol ketosis or soft drink ketosis. Her lactate level was 26 mg/dL, suggesting that lactic acidosis was unlikely.

Since her diabetes diagnosis, the patient had never developed ketosis or ketoacidosis. She had also been taking oral drugs for seven months after her diagnosis. The patient likely had residual insulin secretion at the time of the diagnosis of diabetes mellitus, and her insulin secretion gradually decreased. The urinary C-peptide level was undetectable on admission, suggesting that endogenous insulin secretion gradually decreased and became depleted. The authors therefore believe that the patient had latent autoimmune diabetes in adults.

On admission, she had an elevated glycated albumin level of 27.7% and an HbA1c level of 8.0%, suggesting that the blood glucose level rapidly increased with the administration of the COVID-19 vaccine approximately three days before the onset of ketoacidosis. The clinical presentation of this patient was consistent with ketoacidosis.

This patient likely had a gradual decrease in insulin secretion. She did not develop ketosis or ketoacidosis, even after receiving SGLT-2 inhibitors for one year and four months before admission. In Japan, SGLT-2 inhibitors have been approved for prescription for type 1 diabetic patients since December 2018.

Since SGLT-2 inhibitors can cause metabolic imbalance, the administration of SGLT-2 inhibitors significantly increases the incidence of diabetic ketoacidosis in patients with type 1 diabetes (6). SGLT-2 inhibitors also reportedly increased endogenous glucose production, serum glucagon levels (7), and serum ketones (8). Although, the patient’s carbohydrate and fluid intake in their diet prevented the development of ketosis. In fact, the urinary ketone body was negative at the time of examination two days before the COVID-19 vaccination, and her dietary intake was good.

The administration of the COVID-19 vaccine caused nausea and vomiting, but not ketosis (9). However, nausea and vomiting led to the patient becoming unable to consume carbohydrates. Thus, SGLT-2 inhibitor treatment was discontinued, but her reduced carbohydrate intake and lack of insulin action resulted in ketosis. Ketosis exacerbates nausea, while dehydration exacerbates ketosis, further inducing nausea and vomiting. This vicious cycle resulted in ketoacidosis. The patient also had ketoacidosis, which was complicated by an anaerobic metabolism in the tissues due to rapid metabolic ataxia, probably due to dehydration.

The second case followed a similar course after vaccination without taking SGLT2 inhibitors. Therefore, in addition to the effect of insulin discontinuation as the cause of diabetic ketoacidosis, the effect of the vaccine itself must be examined. We considered the possibility of a temporary decrease in insulin secretion after vaccination common to both patients. And we suspect that is the reason why diabetic ketoacidosis occurred in latent autoimmune diabetes in adults, not acute onset type 1 diabetes.

COVID-19 is known to be a virus that infiltrates and infects cells *via* angiotensin-converting enzyme 2 (ACE2). ACE2 receptors are expressed in various metabolic tissues, including pancreatic beta cells (10) There are also reports of observations of islet cell degeneration in postmortem COVID-19 patients (11). With viral endocytosis, the activation of the renin-angiotensin system through the downregulation of the ACE2 receptor can impair insulin receptor signaling (12). It has also been reported that a similar reaction in islet cells may occur temporarily upon SARS-CoV-2 antigen presentation after vaccination against COVID-19 (13).

To address the COVID-19 pandemic, vaccines have been administered worldwide. In Japan, by December 26, 2021, approximately 77.8% of total population has received the vaccine twice. The percentage of people who have finished vaccination with a history of diabetes is unknown.

A recent meta-analysis confirmed a strong relationship between COVID-19 severity and blood glucose levels (14). Patients with diabetes have a higher risk of severe COVID-19 infection (15) and benefit from the COVID-19 vaccine. It has already been reported that SARS-CoV-2 replication occurs in human beta cells (16).

On the other hand, it has been pointed out that hyperglycemia occurs 1-6 days after the administration of the COVID-19 vaccine in patients with type 2 diabetes. The frequency of hyperglycemia is unknown, but it has been shown to occur soon after vaccination (17). In the United States, case of non-diabetic patient who developed a hyperosmolar hyperglycemic state 6 days after the second vaccination with an mRNA-based vaccine (BNT162b2) have been reported. This patient had an HbA1c value of 5.6% before vaccination, indicating no overt diabetes (18).

Recently, serious adverse events such as thrombosis and cardiomyopathy after COVID-19 vaccination have been reported (19, 20). Similarly, we need to consider the direct link between COVID-19 vaccination and acute hyperglycemic crisis. Because inflammatory cytokine responses from SARS-CoV-2 proteins due to SARS-CoV-2 infection or vaccination can contribute to direct damage to pancreatic islet cells and impaired insulin receptor signaling.

Decreased insulin secretion can induce diabetic ketoacidosis. Diabetic ketoacidosis is generally known to be a major cause of increased hospitalization and mortality in diabetic patients (21). But educational programs can reduce the frequency of ketoacidosis in patients with type 1 diabetes (22). In addition, compliance with the sick day rule is important for preventing the aggravation of ketoacidosis and reducing the frequency and length of hospital stay. Many people with type 1 diabetes say they are confident in managing their physical condition during sick days. However, it is known that the percentage of those who actually take actions, such as increasing their water intake, adjusting their insulin dose, and measuring urinary ketone bodies is small (23). All physicians treating patients with type 1 diabetes must seek ways to encourage their patients to comply with sick day rules. Diabetic patients treated as type 2 diabetics may include type 1 diabetics who have never had diabetic ketoacidosis before. It is important to check the sick day rule in diabetic patients, even when administering the COVID-19 vaccination. We hope that enlightenment activities will be expanded more than ever to reduce deaths and hospitalizations due to diabetic ketoacidosis.

## Conclusion

We propose the following hypothesis to explain the development of diabetic ketoacidosis in the present cases. First, the patients were unable to ingest water and sugar in response to the loss of appetite that occurred after vaccination. Second, despite a decrease in insulin secretion or an increase in insulin requirement, the patients were not to possible perform insulin injection appropriately.

Education from healthcare professionals is important for self-management of diabetes. However, caution is required when social problems are present, especially with when associated with reduced adherence. For such patients, it may be necessary to implement a system where the attending physician can be involved in management during sick days, in addition to self-management.

The two cases presented in the present report involved type 1 diabetes; however, temporary pancreatic β-cell hypofunction may have progressed due to the COVID-19 vaccine itself or as a result of the protein-induced inflammation that occurs after vaccination.

The COVID-19 infection situation is still not stable worldwide, and the occurrence of breakthrough infections after vaccination has become a problem. In Japan, there is an active movement to start the third vaccination in 2021, mainly for elderly people and medical personnel. It is expected that similar cases will increase with increased vaccination coverage. The vaccine should be carefully administered to type 1 diabetes patients receiving intensive insulin therapy and sodium-glucose transporter treatment, as they are at high risk for the development of ketoacidosis.

## Data Availability Statement

The original contributions presented in the study are included in the article/**Supplementary Material**. Further inquiries can be directed to the corresponding author.

## Ethics Statement

Ethical review and approval was not required for the study on human participants in accordance with the local legislation and institutional requirements. The patients/participants provided their written informed consent to participate in this study. Written informed consent was obtained from the individual(s) for the publication of any potentially identifiable images or data included in this article.

## Author Contributions

FY and TM designed the project. FY, TK, and MS obtained written informed consent from the patients. FY and SO clinically characterized the patient and collected clinical information. FY and TM wrote the manuscript. MS, AH, HA, YH, TK, NAw, NAs, TA, SO, and AO were major contributors to the editing of the manuscript. All authors contributed to the article and approved the submitted version.

## Conflict of Interest

The authors declare that the research was conducted in the absence of any commercial or financial relationships that could be construed as a potential conflict of interest.

## Publisher’s Note

All claims expressed in this article are solely those of the authors and do not necessarily represent those of their affiliated organizations, or those of the publisher, the editors and the reviewers. Any product that may be evaluated in this article, or claim that may be made by its manufacturer, is not guaranteed or endorsed by the publisher.
