# Supplementary material for: A Case Series of Ketoacidosis After Coronavirus Disease 2019 Vaccination in Patients With Type 1 Diabetes
Source: Front Endocrinol (Lausanne). 2022 Mar 18;13:840580. doi: 10.3389/fendo.2022.840580 (PMC8971718; doi:10.3389/fendo.2022.840580)
Supplement: Supplementary file 1 [file Presentation_1.pdf]

Supplemental Fig. 1.

**Glucagon load test**		
time	0 min	6 min
Glucose	121 mg/dL	135 mg/dL
C-peptide	<0.03 ng/mL	<0.03 ng/mL

Supplemental Fig legend

Supplemental Fig. 1. Glucagon load test (First Case)

C-peptide was below the limit of detection in a glucagon load test

C-peptide : serum C-peptide immunoreactivity
